# A Novel Fibroblast Growth Factor-1 (FGF1) Mutant that Acts as an FGF Antagonist

**DOI:** 10.1371/journal.pone.0010273

**Published:** 2010-04-21

**Authors:** Satoshi Yamaji, Jun Saegusa, Katsuaki Ieguchi, Masaaki Fujita, Seiji Mori, Yoko K. Takada, Yoshikazu Takada

**Affiliations:** Department of Dermatology, University of California Davis School of Medicine, Sacramento, California, United States of America; Karolinska Institutet, Sweden

## Abstract

**Background:**

Crosstalk between integrins and FGF receptors has been implicated in FGF signaling, but the specifics of the crosstalk are unclear. We recently discovered that 1) FGF1 directly binds to integrin αvβ3, 2) the integrin-binding site and FGF receptor (FGFR) binding site are distinct, and 3) the integrin-binding-defective FGF1 mutant (R50E) is defective in inducing FGF signaling although R50E still binds to FGFR and heparin and induces transient ERK1/2 activation.

**Principal Findings:**

We tested if excess R50E affect DNA synthesis and cell survival induced by WT FGF1 in BaF3 mouse pro-B cells expressing human FGFR1. R50E suppressed DNA synthesis and cell proliferation induced by WT FGF1. We tested if WT FGF1 and R50E generate integrin-FGF1-FGFR ternary complex. WT FGF1 induced ternary complex formation (integrin-FGF-FGFR1) and recruitment of SHP-2 to the complex in NIH 3T3 cells and human umbilical endothelial cells, but R50E was defective in these functions. It has been reported that sustained ERK1/2 activation is integrin-dependent and crucial to cell cycle entry upon FGF stimulation. We thus determined the time-course of ERK1/2 activation induced by WT FGF1 and R50E. We found that WT FGF1 induced sustained activation of ERK1/2, but R50E was defective in this function.

**Conclusions/Significance:**

Our results suggest that 1) R50E is a dominant-negative mutant, 2) Ternary complex formation is involved in FGF signaling, 3) The defect of R50E to bind to integrin may be directly related to the antagonistic action of R50E. Taken together, these results suggest that R50E has potential as a therapeutic in cancer.

## Introduction

Fibroblast growth factors (FGFs) constitute a family of heparin-binding polypeptides involved in the regulation of biological responses, such as growth, differentiation, and angiogenesis [1]–[4]. The biological effects of FGFs are mediated by four structurally related receptor tyrosine kinases denoted FGFR1, FGFR2, FGFR3, and FGFR4. The binding of FGF to its receptor results in receptor dimerization and subsequent transphosphorylation of specific tyrosine residues within the intracellular domain [1]–[4]. Activation of the receptor allows proteins containing Src homology-2 (SH2) or phosphotyrosine binding (PTB) domains to bind to sequence recognition motifs in the FGFR, resulting in phosphorylation and activation of these proteins [5]. This leads to the activation of intracellular signaling cascades. The main signaling cascade activated through the stimulation of FGFR is the Ras/MAP kinase pathway.

Since FGF signaling enhances multiple biological processes that promote tumor progression [6], it is an attractive therapeutic target. This is particularly important because therapies targeting FGF receptors and/or FGF signaling not only affect the growth of the tumor cells but also modulate tumor angiogenesis [7]. FGF1 and FGF2 are also pro-inflammatory growth factors [8] that play a role in pathological angiogenesis in chronic inflammatory diseases. Thus FGF signaling is a potential therapeutic target for pathological angiogenesis in chronic inflammatory diseases.

Integrins are a family of cell adhesion receptors that recognize extracellular matrix (ECM) ligands and cell surface ligands [9]. Integrins are transmembrane αβ heterodimers, and at least 18 α and 8 β subunits are known [10]. Integrins play an important role in anchorage-dependent cell survival and proliferation [11]. Integrins transduce signals to the cell upon ligand binding, and their functions are in turn regulated by the signals from within the cell [9]. Ligation of integrins triggers a large variety of signal transduction events that serve to modulate cell behaviors including proliferation, survival/apoptosis, shape, polarity, motility, gene expression, and differentiation.

Recently we reported that FGF1 directly bound to integrin αvβ3 and localized the integrin-binding site in FGF1 within or close to the heparin-binding site, but distinct from the FGFR-binding site [12]. An FGF1 mutant (the Arg-50 to Glu mutant, R50E) was defective in binding to αvβ3, but still bound to FGFR or heparin. We showed that R50E was defective in inducing DNA synthesis, cell proliferation, and migration, while it still could induce initial FGFR1 phosphorylation, FRS2α phosphorylation and ERK1/2 phosphorylation [12]. We hypothesized that the direct binding of FGF1 to αvβ3 is a potential mechanism for FGFR-integrin crosstalk. We predict that the defect of R50E is located in the later steps of FGF signaling, and that R50E is a useful tool for studying the role of integrins in FGF signaling.

In the present study, we demonstrate that the R50E mutant of FGF1 suppressed FGF signaling induced by WT FGF1 in a dominant-negative fashion. We studied the mechanism of the antagonistic action of R50E. R50E induced transient ERK1/2 activation, but did not induce sustained ERK1/2 activation, which is integrin-dependent and directly related to cell cycle entry [13], [14]. We found that WT FGF1 induced the FGFR-FGF-integrin ternary complex formation, but R50E did not. Our results suggest that R50E could not bring αvβ3 to FGFR due to defective integrin-FGF interaction, and thereby disrupted subsequent signaling steps. Thus integrin-FGF interaction plays a critical role in FGF signaling and represents a novel therapeutic target.

## Results

### Dominant-negative effect induced by R50E

**Figure 1:**
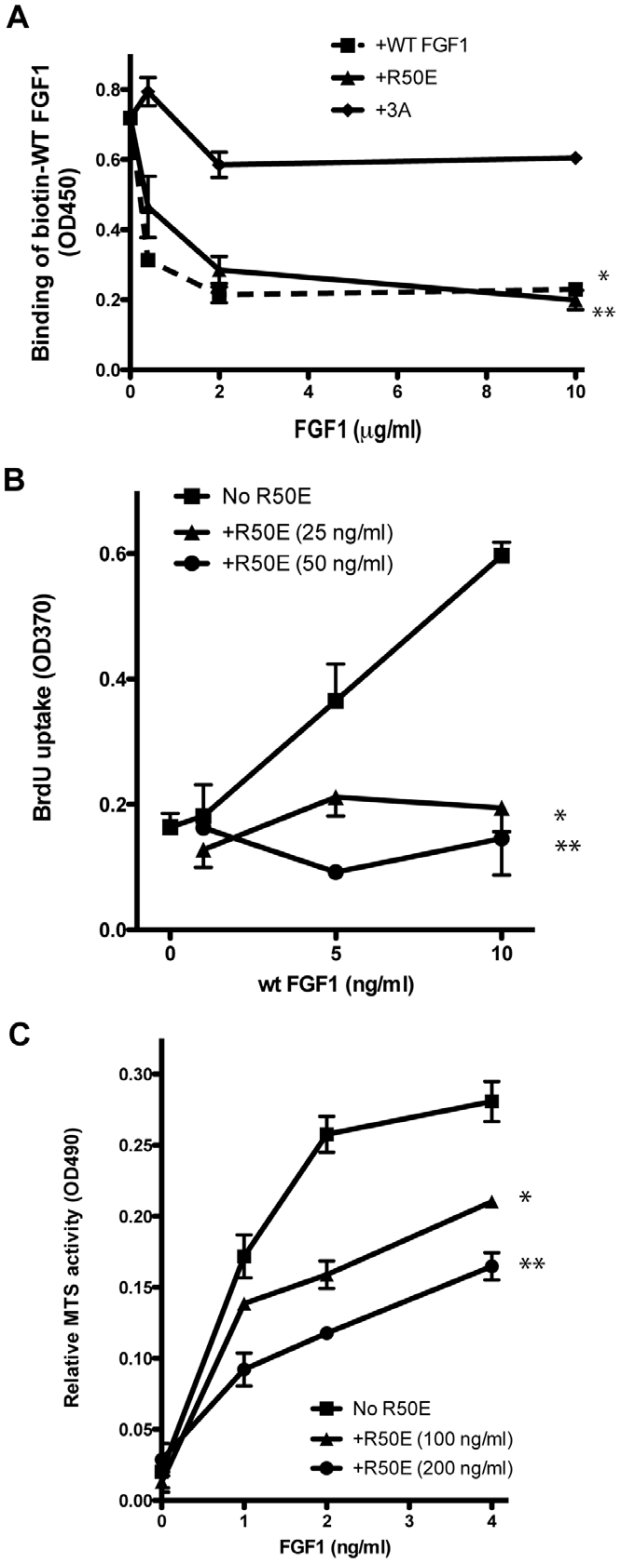
R50E is a dominant-negative inhibitor of FGF signaling. A.R50E competed with WT FGF1 for binding to the FGFR1 D2D3 fragment. Biotinylated FGF1 and increasing concentrations of unlabelled FGF1 or FGF1 mutants were incubated with the immobilized FGFR1 D2D3 fragment and bound biotinylated FGF1 was measured with HRP-conjugated avidin. The 3xA mutation located at the predicted FGF-FGFR binding site [1] was used as a negative control. The results indicate that R50E competitively blocked the binding of biotinylated WT FGF1 to FGFR fragment to the same extent as WT FGF1. * P<0.0001, ** P = 0.0002 (n = 3) compared to +3A. There is no significant difference between WT and R50E at 10 µg/ml. B. R50E suppressed the DNA synthesis in BaF3-FGFR1c cells induced by WT FGF1. We cultured BaF3-FGFR1c cells in the presence of 1 ng/ml WT FGF1 and 25 or 50 ng/ml R50E for 24 h instead of IL-3 and measured incorporation of BrdU. Results are shown as means +/−SEM. * P<0.0001, ** P = 0.0003 by t-test (n = 4) compared to No R50E. C. R50E suppressed the proliferation of BaF3-FGFR1c cells induced by WT FGF1. We cultured BaF3-FGFR1c cells in the presence of 1 ng/ml WT FGF1 and 100 or 200 ng/ml R50E for 24 h instead of IL-3 and measured cell proliferation by MTS assays. Results are shown as means +/− SEM. * P<0.0025, ** P = 0.0093 by t-test (n = 3) compared to No R50E.

Our previous study shows that integrin-binding-defective R50E is defective in inducing DNA synthesis, chemotaxis, and cell proliferation, while it can bind to FGFR1 and heparin [12]. These results suggest that FGF1 binding to integrins plays a role in FGF signaling. If FGF1 needs to bind to both FGFR and integrins for signaling, it is predicted that R50E that cannot bind to αvβ3 is not only defective in signaling but suppresses signaling induced by WT FGF1 in a dominant-negative manner. To assess this hypothesis, we first tested if R50E competes with biotinylated WT FGF1 for binding to the immobilized FGFR D2D3 fragment in ELISA-type binding assays. We used the 3xA mutant that is defective in binding to FGFR1 [12] as a control. We found that unlabeled R50E effectively reduced the binding of biotinylated WT FGF but the 3xA mutant did not (Fig. 1A). These results suggest that R50E competitively blocked the binding of WT FGF1 to FGFR1, which is consistent with our previous results that WT FGF1 and R50E have similar affinity to FGFR1 using surface plasmon resonance [12]. We next tested if R50E suppresses cell proliferation in a competitive manner. BaF3 mouse pro-B cells that express human FGFR1c (designated BaF3-FGFR1c) have been widely used to sensitively detect cell proliferation that is dependent on exogenous FGF [15]. We reported that WT FGF1 induced proliferation of BaF3-FGFR1c cells, but R50E did not [12]. We found that excess R50E blocked WT FGF1-induced BrdU incorporation in BaF3-FGFR1c in a dose-dependent manner (Fig. 1B), suggesting that R50E acts as a dominant-negative antagonist of FGF1-induced DNA synthesis. We next tested if R50E affects cell proliferation induced by WT FGF1 using MTS assays. We found that excess R50E blocked WT FGF1-induced BaF3-FGFR1c cell proliferation in a dose-dependent manner (Fig. 1C), suggesting that R50E acts as a dominant-negative antagonist of FGF1-induced cell proliferation. Taken together, our results suggest that R50E is a novel competitive inhibitor of FGF signaling.

### WT FGF1 induces ternary complex formation (FGFR1, FGF1, and integrin β3) while R50E is defective in this function

**Figure 2:**
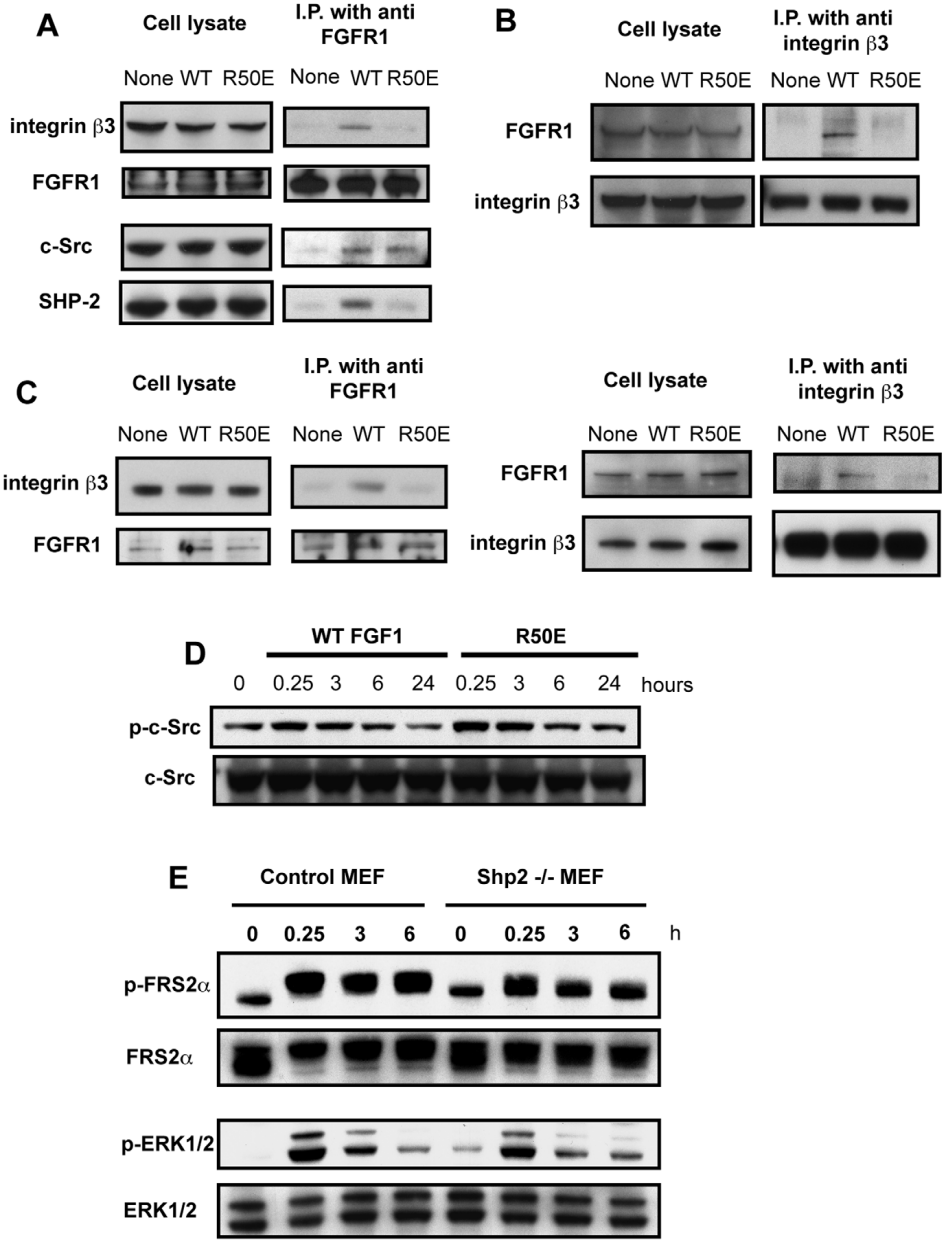
WT FGF1 induced FGFR1-FGF-αvβ3 ternary complex formation, but R50E was defective in this function. We immunoprecipitated the FGFR1-αvβ3 complex from cell lysates with anti-FGFR1 (A) or anti-β3 mAb (B), and analyzed the immuno-purified materials by Western blot analysis. A. WT FGF1 induced co-immunoprecipitation of integrin β3 and SHP-1 with FGFR1 using anti-FGFR1, but R50E was defective in this function. B. WT FGF1 induced co-immunoprecipitation of FGFR1 with integrin β3 using anti-integrin β3, but R50E was defective in this function. We stimulated serum-starved NIH 3T3 cells with 5 ng/ml WT FGF1 or R50E for 1 h in the presence of 5 µg/ml heparin. C. Co-precipitation of integrin β3 and FGFR1 upon FGF1 stimulation in HUVEC. Serum-starved HUVEC were stimulated by 5 ng/ml WT FGF1 or R50E with 5 µg/ml heparin for 1 h. Cell lysates were immunoprecipitated with anti-FGFR1 or anti-β3 monoclonal antibody, and the immunoprecipitates were analyzed by Western blotting. D. WT FGF and R50E similarly activated c-Src. We stimulated serum starved NIH 3T3 cells with WT FGF1 or R50E, and cell lysates were analyzed by Western blotting using antibodies specific to phospho-c-Src or c-Src. E. Time-course of embryonic fibroblasts from SHP-2 (−/−) or control mice. Serum starved cells were treated with WT FGF1 or R50E (5 ng/ml) for the time indicated and cell lysates were analyzed by Western blotting.

If the integrin αvβ3-binding site in FGF1 is distinct from that of FGFR1 [12], FGF1 can simultaneously bind to αvβ3 and FGFR (ternary complex formation). To directly demonstrate the ternary complex, we stimulated the serum-starved NIH 3T3 cells with FGF1 for 1 h and immuno-precipitated FGFR1 from cell lysates using anti-FGFR1 mAb, and analyzed the immuno-purified materials by Western blotting with anti-β3 antibody. We detected integrin β3 in the immune complex when WT FGF1 was used but did not detect integrin β3 when R50E was used (Fig. 2A). This suggests that the ternary complex formation depends on the integrin-binding function of FGF1. As a reciprocal experiment we used anti-integrin β3 mAb instead of anti-FGFR1 antibody for immuno precipitation, and detected FGFR1 and FGF1 in the immune complex when WT FGF1 was used. However, we did not detect FGFR1 or FGF1 when R50E was used (Fig. 2B). We obtained similar results when cells were incubated for 10 min instead of 1 h (data not shown). Taken together, these results suggest that the simultaneous binding of FGFR1 and integrin αvβ3 to FGF1 occurs during FGF signaling. Notably, the integrin-binding-defective R50E mutant failed to induce ternary complex formation, and this may be related to the dominant-negative effect (Fig. 1).

To test if FGF1 induces the FGF1-αvβ3 complex formation in other cell types, we stimulated the serum-starved HUVEC with wt FGF1 and immunoprecipitated αvβ3 from cell lysates using anti-FGFR1 mAb, and analyzed the immunoprecipitates by Western blotting with anti-β3 antibody. We detected integrin β3 in the immune complex only when wt FGF1 was used (Fig. 2C). As a reciprocal experiment we used anti-integrin β3 instead of anti-FGFR1 for immuno precipitation, and detected FGFR1 in the immune complex upon wt FGF1 stimulation. However, we did not detect the FGFR1-αvβ3 complex in the immunoprecipitates upon R50E stimulation (Fig. 2C). These results suggest that the ternary complex formation and the effect of the R50E mutation are not cell-type specific.

It has been reported that the non-receptor tyrosine kinase Src is recruited to activated fibroblast growth factor receptor (FGFR) complexes through the adapter protein factor receptor substrate 2 (FRS2) [16]. We tested if R50E affects the levels of c-Src activation. There was no difference in the levels of Src activation between WT FGF1 and R50E (Fig. 2D). Also, c-Src is associated with FGFR1 after WT FGF1 and R50E stimulation at comparable levels (Fig. 2A), suggesting that FGF-integrin interaction is not related to the c-Src recruitment to the FGF/FGFR complex.

It has been reported that IGF1 induces recruitment of the SH2 domain-containing protein phosphatase SHP-2 to the tyrosine phosphorylated integrin β3 cytoplasmic domain [17]. Also, FGF2 induces the recruitment of SHP-2 to the FGFR-FRS2α complex and the sustained FRS2α phosphorylation, and these steps are essential for sustained ERK1/2 activation [18], [19]. We tested if the R50E mutation affects recruitment of SHP-2. We detected SHP-2 in the ternary complex when WT FGF1 was used for stimulating NIH 3T3 cells (Fig. 2A). Interestingly, we did not detect SHP-2 in the immunoprecipitate from R50E-stimulated NIH 3T3 cells. These results suggest that WT FGF1, but not R50E, induced the recruitment of SHP-2 to the ternary complex, and that the ternary complex formation and simultaneous recruitment of SHP-2 may be critical in FGF signaling. We also tested if SHP-2 is critical for FGF signaling using mouse embryonic fibroblasts deficient in SHP-2 [20]. The levels of sustained FRS2α and ERK1/2 activity upon WT FGF1 stimulation were lower in mouse embryonic fibroblasts deficient in SHP-2 than control embryonic fibroblasts (Fig. 2E). Taken together our results suggest that recruitment of SHP-2 to the FGF/FGFR/integrin ternary complex is required for FGF signaling.

### R50E is defective in inducing sustained ERK1/2 activation

**Figure 3:**
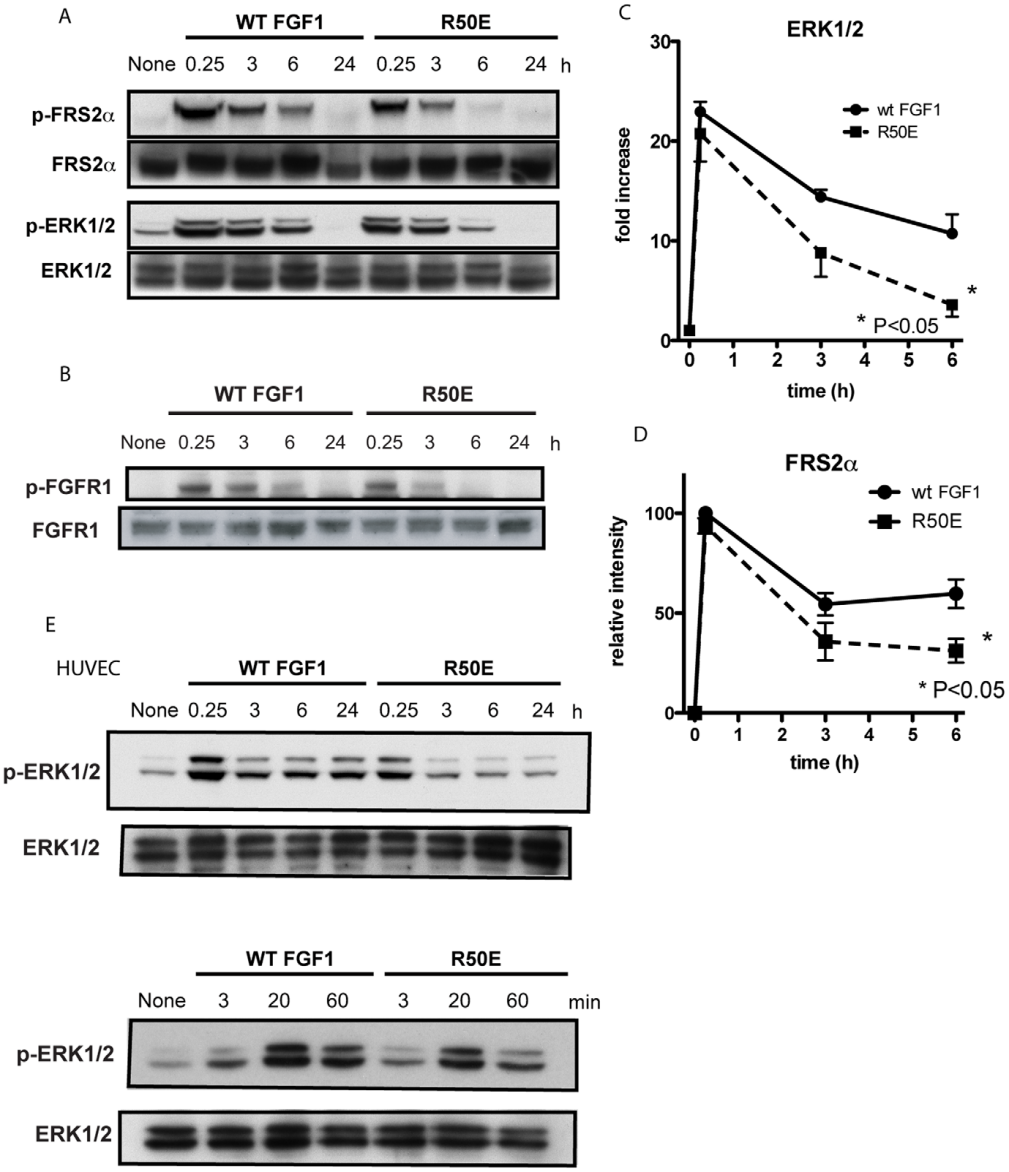
R50E is less effective in inducing sustained ERK1/2 activation in NIH3T3 cells. A. Time-course of ERK1/2 activation and FRS2α activation induced by WT FGF1- or R50E-stimulated cells. We stimulated serum-starved cells with 5 ng/ml WT FGF1 or R50E at indicated time points and analyzed cell lysates by Western blotting using anti phospho-FRS2α, phospho-ERK1/2, total-FRS2α, or ERK1/2 antibody. A representative data is shown from several independent experiments. B. Time-course of WT FGF1 or R50E induced FGFR1 phosphorylation. We stimulated serum starved cells with 5 ng/ml WT FGF1 or R50E in the presence of 5 µg/ml heparin, and analyzed cell lysates by Western blotting. A representative data is shown from several independent experiments. C. ERK1/2 activation levels more rapidly reduced in cells stimulated with R50E than cells stimulated with WT FGF1. Lysates of cells stimulated with R50E or WT FGF1 were analyzed by Western blotting using anti phospho- or total FRS2α antibody. Fold increase of ERK1/2 signals (phosphorylated protein/total protein) is shown with control “time 0” as 1. Data are shown as means +/− SEM of triplicate experiments. ERK1/2 activation at 6 h with R50E is significantly lower in than with WT FGF1 (* P<0.05, n = 3). D. FRS2α phosphorylation levels reduced more rapidly in cells stimulated with R50E than in cells stimulated with WT FGF1. Lysates of cells stimulated with R50E or WT FGF1 were analyzed by Western blotting using anti phospho- or total FRS2α antibody. Relative intensity of FRS2α signals is shown with density at 15 min as 100. The level of total FRS2α in each lane was comparable using anti-FRS2α (data not shown). FRS2α phosphorylation at 6 h with R50E is significantly lower in than with WT FGF1 (* P<0.05, n = 3). E. Time-course of ERK1/2 activation induced by WT FGF1-or R50E-stimulated human umbilical endothelial cells (HUVECs). Experiments were performed as described in A except that HUVEC was used.

The present results suggest that R50E is defective in inducing ternary complex formation because it is defective in direct binding to integrins [12]. What is the consequences of the defective ternary complex formation? It has been reported that sustained ERK1/2 activation is integrin-dependent [13] and crucial to cell cycle entry [14] upon FGF stimulation. We thus hypothesized that R50E is defective in inducing sustained ERK1/2 activation. We found that R50E induced transient ERK1/2 activation to the extent similar to that of WT FGF1 (until 3 h after stimulation), but could not maintain high ERK1/2 levels after 6 h in NIH 3T3 cells (Fig. 3A and 3C). Also, FRS2α phosphorylation (Fig. 3A and 3D) and FGFR1 phosphorylation (Fig. 3B) followed the time-course similar to that of ERK1/2. Both WT FGF1 and R50E induced FRS2α and FGFR1 phosphorylation in 15 min, but FRS2α and FGFR1 phosphorylation more rapidly reduced in 6 h with R50E than with WT FGF1. We obtained similar results with HUVEC (Fig. 3E): transient ERK1/2 activity decreased more quickly when cells were treated with R50E than with WT FGF1. These results show that the direct binding of integrins to FGF1 is required for sustained ERK1/2 activation. Since R50E induced transient activation of FGFR1, FRS2α, and ERK1/2 [12], it is suggested that transient induction of FGF signaling does not require the direct binding of FGF to integrins. These findings suggest that the direct binding of integrins to WT FGF1 plays a role in sustained activation of the entire FGF signaling pathway.

## Discussion

In the present study we establish that the integrin-binding-defective R50E mutant of FGF1 is a dominant-negative antagonist of FGF1. We demonstrated that excess R50E suppressed DNA synthesis and cell proliferation induced by WT FGF1. Because FGF1 binds to all known FGFR isoforms [15], it is expected that R50E will block signaling by other members of the FGF family.

It has been reported that receptor binding of heparin-binding growth factors (HB-GFs), such as FGF, is regulated by interactions with heparan sulfate proteoglycans [21]. We demonstrated that the R50E mutation, however, did not affect heparin-FGF1 interaction in surface plasmon resonance study [12]. Thus it is unlikely that the effect of the R50E mutation on FGF signaling is explained by its effect on FGF-heparin interaction. The presence of heparin in the experimental system is unlikely to affect the effect of the R50E mutation on FGF signaling. It is also unlikely that the effect of the R50E mutation on FGF signaling is due to its effect on FGFR binding, since we showed that R50E binds to FGFR1 at levels comparable to that of WT FGF1 in competitive binding assays (this study) and in surface plasmon resonance studies [12].

We studied the mechanism of the dominant-negative effect of R50E. We presented evidence that WT FGF1 co-precipitation of integrin αvβ3 and FGFR1, and recruitment of SHP-2 to the complex, while R50E was defective in these functions. Since R50E can still bind to FGFR1 and heparin [12], it is likely that the defect in integrin binding is related to its antagonistic action in FGF signaling, and that crosstalk between integrins and FGFR is mediated by the direct binding of integrins to FGF. Since R50E cannot bring integrin αvβ3 to the FGF/FGFR complex, R50E effectively interrupts the subsequent FGF signaling events. It has been recently reported that WT FGF2 induced co-precipitation of αvβ3 and FGFR in HMVEC [22]. Thus it is possible that FGF2 induces intracellular signals through ternary complex formation.

It has been established that the duration of ERK pathway signaling determines the proliferative response of cells [14]. The induction of cell cycle re-entry requires sustained ERK signaling and the subsequent activation of successive waves of gene expression, culminating in a set of proliferative genes that includes cyclin D1 [14]. FGF causes sustained ERK pathway activation and promotes proliferation. cyclin D1 expression is not activated by transient ERK signaling but is only triggered after sustained activation of this pathway [23], [24]. We presented evidence that WT FGF1 maintained ERK1/2 activation after 6 h of stimulation (sustained ERK1/2 activation), while R50E was defective in this function. It has been reported that sustained ERK1/2 activation is integrin-dependent [13] upon FGF stimulation. Upon initiation of angiogenesis with FGF2 on the chick chorioallantoic membrane, endothelial cell ERK activity is detected as early as 5 min yet is sustained for at least 20 h. The transient ERK activity (5–120 min) is refractory to integrin antagonists, whereas the sustained activity (4–20 h) depends on integrin αvβ3, but not β1 integrins [13]. It is thus likely that the ability of R50E to disrupt DNA synthesis and cell proliferation induced by WT FGF1 may be a consequence of its defect in integrin binding and inability thereby to induce sustained ERK1/2 activation. However, how integrins are involved in inducing sustained ERK1/2 activation is unclear at this point.

## Materials and Methods

### Materials

WT FGF1, the R50E mutant of FGF1, and the FGFR1 D2D3 fragment were synthesized as described [12]. Human umbilical endothelial cells (HUVEC) (Cascade Biologics, Portland, OR) were cultured under the same conditions as NIH 3T3 cells (ATCC, Manassas,VA) except for the use of M200 medium supplemented with low serum growth supplement (Cascade Biologics) instead of DMEM containing10% FCS. HUVEC were used between passage 3 and 6. Embryonic fibroblasts from SHP-2 deficient mice were kindly provided by Gen Sheng-Feng (Burnham Institute, La Jolla, CA). The antibodies were obtained from the following sources: anti-polyclonal FGFR1 from Abgent (San Diego, CA); anti-phospho FGFR1 (Tyr-653/Tyr-654) from Biosource (Camarillo, CA); anti-FRS2α, and anti-FGF1 from Santa Cruz Biotechnology (Santa Cruz, CA); anti-phospho-FRS2α (Tyr-196), anti-p44/42 MAPK (ERK1/2), anti-phospho-p44/42 MAPK (ERK1/2) (Thr-202/Tyr-204), anti-integrin polyclonal integrin β3, anti-c-Src, anti-phospho-c-Src (Tyr-416), and anti-SHP-2 antibodies from Cell Signaling Technology (Danvers, MA).

### Methods

#### Western blot analysis

NIH 3T3 cells or HUVEC were grown to 70–80% confluence, and starved in DMEM supplemented with 0.4% FCS for 16 h. The cells were then treated with wild type (WT) or mutant FGF1 (5 ng/ml) in the presence of 5 µg/ml heparin for 15 min–24 h at 37°C. Then, cells were washed twice with ice-cold PBS, and lysed with the lysis buffer (PBS containing 1% NP−40, 0.5% Sodium deoxycholate, 0.1% SDS, protein inhibitor cocktail (Sigma), 1 mM PMSF, 20 mM NaF, and 1 mM Na_3_VO_4_.). Protein concentrations in the cell lysates were determined using the BCA protein assay kit (Thermo Scientific). Equal amounts of proteins were analyzed by SDS-PAGE in a 4–12% polyacrylamide gel and standard Western blot analysis protocol. Bound antibodies were detected with HRP-conjugated anti-mouse or anti-rabbit IgG (BioRad), and Super Signal WestPico (Thermo Scientific). Blots were imaged and quantified by Multi Gauge V3.0 software (Fuji film Co.).

#### Immunoprecipitation

Cells cultured in 10-cm dishes were suspended in 300 µl of lysis buffer containing 20 mM Hepes, pH 7.4, 100 mM NaCl, 10% glycerol, 1 mM MgCl_2_, protease inhibitor cocktail (Sigma), 1 mM PMSF, 20 mM NaF, 1 mM Na_3_VO_4_ and 1.0% NP-40. After a 30-min incubation on ice, we clarified the lysates by centrifugation at 14,000 rpm for 20 min and adjusted the protein concentration to 2.5 mg in 750 µl lysis buffer, then incubated with 20 µl of protein G-Sepharose (Invitrogen) conjugated with 1.5 µg of anti-FGFR1 (M2F12) mAb (Santa Cruz), 5 µg of rat anti-integrin β3 mAb (MBL), or mouse anti human integrin β3 mAb (AV10) for 24 h at 4°C. After washing with the same lysis buffer except for 0.5% NP-40 three times, we solubilized the immune complex by adding SDS sample buffer to the resin and analyzed by Western blot analysis.

#### Competitive binding of WT and mutant FGF1 to the FGFR1 D2D3 fragment

The FGFR D2D3 fragment, which contains the FGF-binding site, and the 3xA mutant of FGF1 were prepared as described [12]. We coated wells of 96-well microtiter plates with (100 µl PBS containing 1 µg/ml FGFR D2D3) for overnight at 4°C, and the remaining protein binding sites were blocked with BSA. We then added 100 µl Tyrode-HEPES buffer that contains biotinylated WT FGF1 (1 µg/ml) and non-labeled WT FGF1 or mutant FGF1 (0–10 µg/ml) and incubated for 3 h at room temperature. The 3xA FGF1 mutant that is defective in FGFR binding site [12] was used as a control. After extensive washing with Tyrode-HEPES buffer, we determined bound biotinylated WT FGF1 using HRP-conjugated streptavidin and peroxidase substrate (3,3′,5,5′-tetramethylbenzidine, TMB).

#### DNA synthesis and proliferation of BaF3 cells that express human FGFR1c isoform

We maintained mouse pro B BaF3 cells that express human FGFR1c (BaF3-FGFR1c, kindly provided by David Ornitz, Washington University, St. Louis, MO) in a medium containing 0.5 ng/ml IL-3 as described [15]. DNA synthesis was measured by BrdU assays. Briefly, 20,000 cells per well were incubated with WT or R50E FGF instead of IL-3 at 37°C for 24 h in a 96-well assay plate in a RPMI medium containing 0.5% FCS and 2 µg/ml heparin. Cells were then incubated with BrdU for 4 h. Incorporated BrdU were measured by cell proliferation ELISA kit (Roche) at 370 nm. For proliferation assays, cells were maintained with WT or mutant FGF1 instead of IL-3. Cell proliferation was assessed based on the ability of the cells to convert MTS (3-(4,5-dimethylthiazol-2-yl)-5-(3-carboxymethoxyphenyl)-2-(4-sulfophenyl)-2H-tetrazolium) into formazan, using the Aqueous Cell Proliferation Assay Kit (Promega, Madison, WI). Cells were plated in 96-well plates (1×10^4^ cells/well), and then incubated with DMEM containing 10% FBS at 37°C in 5% CO_2_ atmosphere. Twenty µl of MTS reagent was added to each well at the indicated time period. Relative cell number was measured based on increased absorbance at 490 nm.

#### Statistical analysis

Statistical analysis was performed using Prism software (GraphPad software).
